# Supplementary figures and images for: Assessment of antibody library diversity through next generation sequencing and technical error compensation
Source: PLoS One. 2017 May 15;12(5):e0177574. doi: 10.1371/journal.pone.0177574 (PMC5432181; doi:10.1371/journal.pone.0177574)

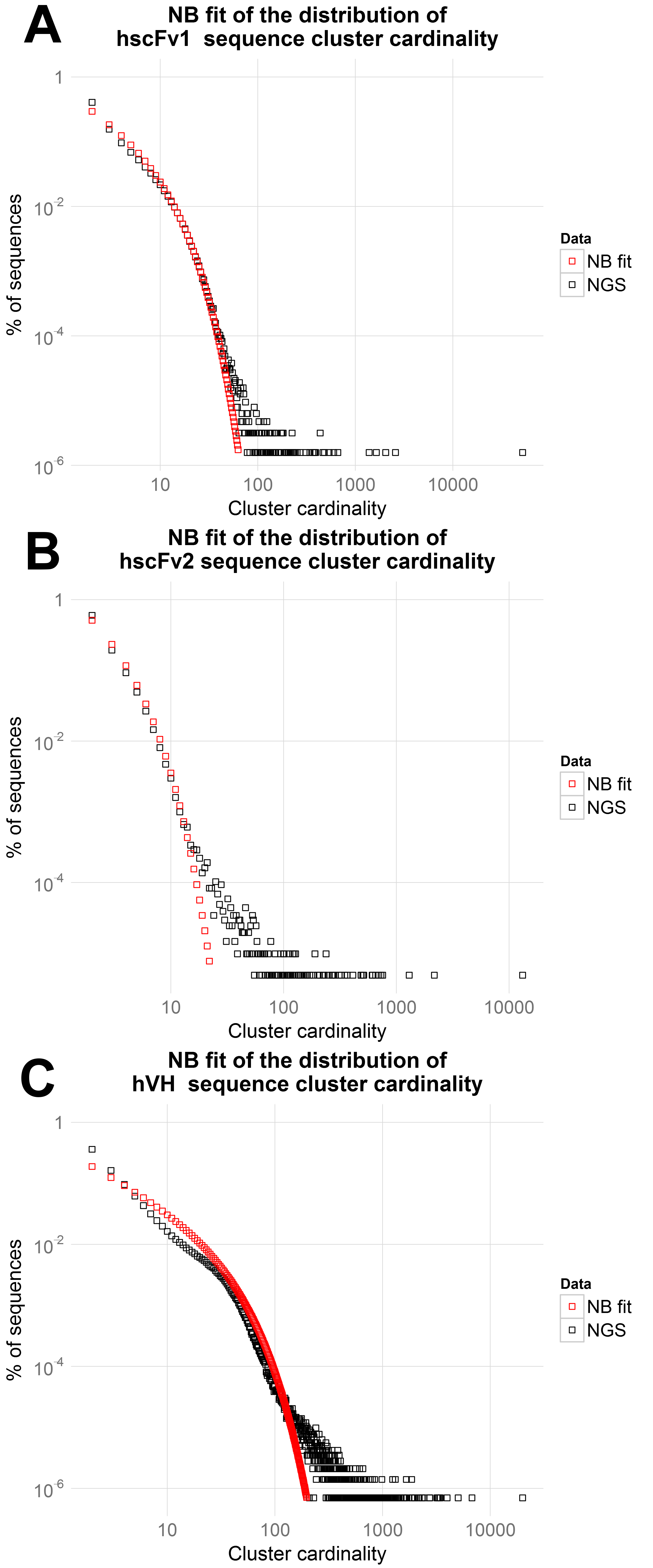

Supplement: S3 Fig — Distribution of library sequence cluster cardinality and regression curve. The three library A) hscFv1, B) hscFv2 and C) hVH (in black) are plotted with the corresponding Negative Binomial regression fit (in red). (TIF) [file pone.0177574.s003.tif]

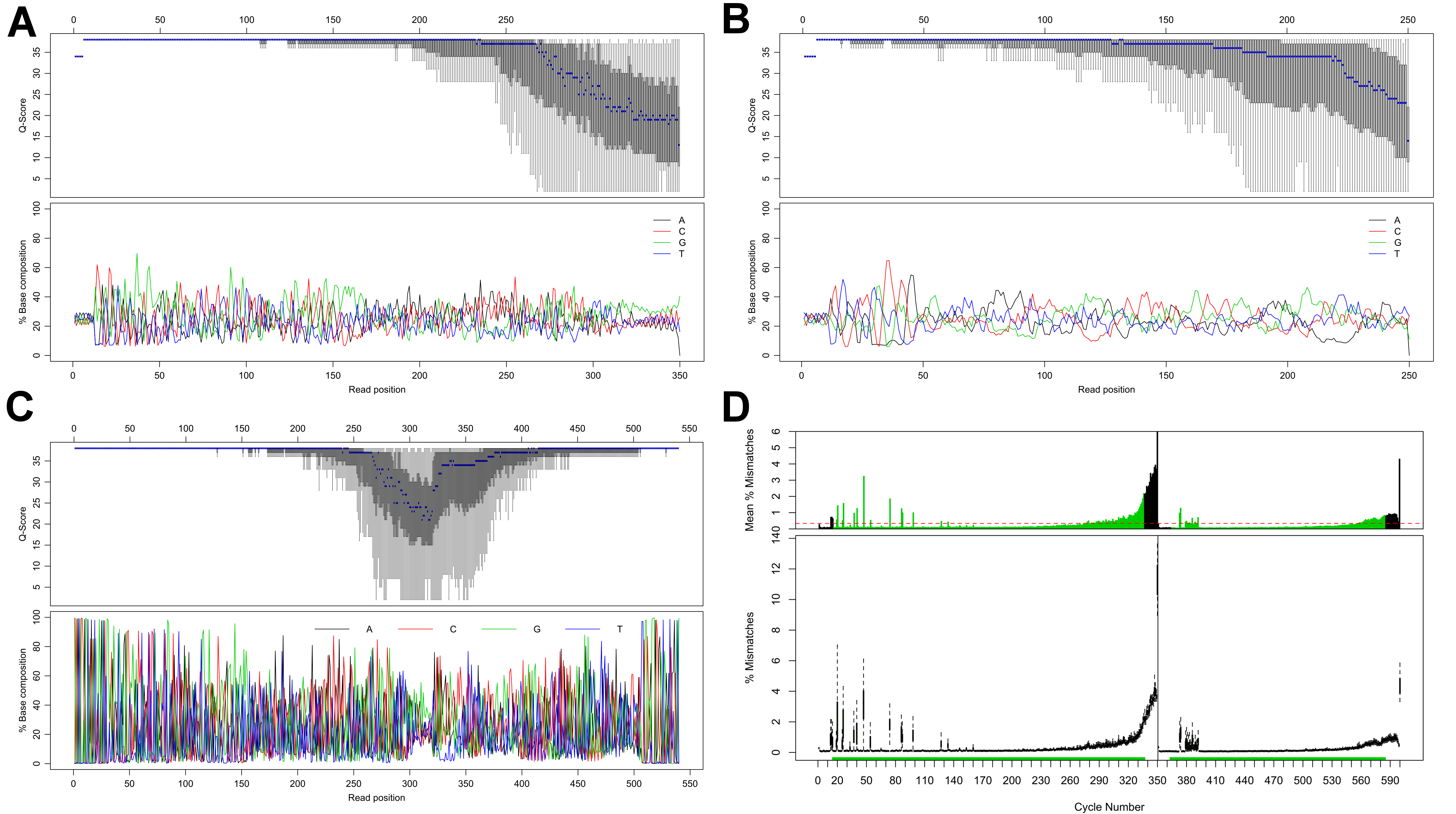

Supplement: S4 Fig — A) Upper panel: box and whisker plot of R1 Phred quality score per sequencing cycle. Median Phred score remained greater than 30 beyond the 300th cycle. Bottom panel: base composition per cycle. The first dozen bases, critical for cluster detection, are balanced due to index presence. B) Upper panel: box and whisker plot of R2 Phred quality score per sequencing cycle. Median Phred score remained greater than 30 beyond the 200th cycle. Bottom panel: base composition per cycle. The first dozen bases are balanced due to index presence. C) Upper panel: box and whisker plot of joined R1-R2 after index and end trimming Phred quality score of hscFv1. Median Phred score remained greater than 30 in all considered position. Bottom panel: base composition in the considered positions. After index trimming the first and last hundreds bases appear well conserved (belonging to the constant region of variable fragment). D) Phi-X technical error rate per sequencing cycle. Green represent the region after trimming. Upper panel: barplot of the mean %mismatches among sequencing tiles. Bottom panel: box and whisker plot of %mismatches. Error rate is more prominent in the beginning sequencing cycles (spikes), with a small increase at the end of each read. Similar results were obtained for hscFv2 and hVH libraries. (TIF) [file pone.0177574.s004.tif]
