# Supplementary material for: Assessment of antibody library diversity through next generation sequencing and technical error compensation
Source: PLoS One. 2017 May 15;12(5):e0177574. doi: 10.1371/journal.pone.0177574 (PMC5432181; doi:10.1371/journal.pone.0177574)
Supplement: S1 File — Detailed protocol for library construction and statistical analysis. (DOCX) [file pone.0177574.s006.docx]

**Supporting Information**

**Construction of hscFv1 library**

The scFv antibody library was assembled from human lymphocytes extracted from peripheral blood (PBLs) following a protocol modified from Marks and Bradbury [1]. RNA was isolated from 1x10^7^ PBLs of four voluntary donors using Trizol. cDNA of IgM (heavy and light chains) was used as template to amplify VH and VL (both kappa and lambda) regions. Primers were designed to anneal to the external framework regions of the V genes. Construction of the library occurred in five steps as shown in S1 Fig: i) amplification of VHs, Vκs and Vλs from cDNA; ii) Construction of a linker (G4S)3 with primers specific for VHs, Vκs and Vλs; iii) Assembly of each variable class (VHs, Vκs and Vλs) with the specific (G4S)3 linker (VH, Vκ and Vλ blocks); iv) pullthrough of VH block with Vκ blocks and VH blocks with Vλ blocks to generate scFv objects, and addition of restriction sites for BssHII at 5’ and for NheI at the 3’ to the scFv pullthrough products; v) Ligations of BssHII/NheI digested pullthroughs to vector pLinker220 . Briefly, for the first step, 6 primers for the 5’ and 4 primers for the 3’ were used to amplify VHs, 6 primers for the 5’ and 5 primers for the 3’ were used to amplify Vκs and 7 primers for the 5’ and 3 primers for the 3’ were used to amplify Vλs (see list below). Every possible combination of these primers for each V chain was used, generating 24 individual classes of VHs, 30 of Vκs and 21 of Vλs. For the second step, a 45 bp linker (G4S)3 was amplified from a pre-existing plasmid using a set of primers, with the 3’ region annealing on the linker, and different protruding 5’, overlapping perfectly either with VH framework4 or VL framework1. At this point, all classes individually amplified were mixed in equimolar ratio for VHs, Vκs and Vλs, and the mix used in the third step in a PCR to join V products to each V specific linker. The fourth step was performed joining VH blocks and VL blocks in a final pullthrough PCR (in which the linkers overlap) to produce the scFv. In this step the first 10 cycles were run without primers allowing the joining of the different blocks. Then, primers for the 5’ of VHs bearing restriction site for BssHII and primers for the 3’ of Vκs or Vλs bearing restriction site for NheI were added to the mix, generating the final ~750bp scFv product. Finally the pullthrough products were digested with BssHII/NheI enzymes and ligated to the BssHII/NheI digested vector pLinker220 [2].

~1µg of ligation product was transformed by electroporation in Max Efficiency DH5α (Invitrogen). Transformation efficiency was assessed by plating on selective media serial dilution of the transformation and counting surviving colonies. The theoretical maximum of the library complexity was 1.58 x10^7^. Transformed bacteria were inoculated in LB-SeaPrep Agarose (Lonza Rockland, Inc.) as described in Elsaesser [3]. Plasmids were extracted with Qiagen Plasmid Giga Kit.

**Construction of hscFv2 library**

The starting RNA, as well as all the primers in each step, was the same used for hscFV1 library. In the first step of the process, a mix of the 6 primers for the 5’ and of the 4 primers for the 3’ was used in a single reaction to amplify all VH subclasses concomitantly. Similarly, a mix of the 6 primers for the 5’ and of the 5 primers for the 3’ was used to amplify Vκs and a mix of the 7 primers for the 5’ and of the 3 primers for the 3’ was used to amplify Vλs. The subsequent steps of the process were carried out as for the construction of hscFv1. Transformation efficiency was assessed as described before and was 1.4 x10^7^. Transformed bacteria were inoculated in LB-SeaPrep Agarose (Lonza Rockland, Inc.) as described in Elsaesser [3]. Plasmids were extracted with Qiagen Plasmid Giga Kit.

**Construction of hVH library**

The starting RNA was the same used for hscFV1 library. Amplification of VH subclasses was performed using in a single reaction a mix of the 6 primers for the 5’ (bearing BssHII restriction site) and of the 4 primers for the 3’ (bearing NheI restriction site) specific for VH. VH products were digested with BssHII/NheI enzymes and ligated to the BssHII/NheI digested vector pLinker220. Transformation efficiency was assessed as described before and resulted to be 6 x10^6^.Transformed bacteria were inoculated in LB-SeaPrep Agarose (Lonza Rockland, Inc.) as described in Elsaesser [3]. Plasmids were extracted with Qiagen Plasmid Giga Kit.

**Primers used for cDNA**

HuCκFOR: 5’ AGACTCTCCCCTGTTGAAGCTCTT 3’

HuCLFOR: 5’ TGAAGATTCTGTAGGGGCCACTGTCTT 3’

**Primers used for library construction**

Primers for VH

BssHII-HuVH1aBACK: 5’ gCCgcgcgcatgccCAGGTGCAGCTGGTGCAGTCTGG 3’

BssHII-HuVH2aBACK: 5’ gCCgcgcgcatgccCAGGTCAACTTAAGGGAGTCTGG 3’

BssHII-HuVH3aBACK: 5’ gCCgcgcgcatgccGAGGTGCAGCTGGTGGAGTCTGG 3’

BssHII-HuVH4aBACK: 5’gCCgcgcgcatgccCAGGTGCAGCTGCAGGAGTCGGG 3’

BssHII-HuVH5aBACK: 5’ gCCgcgcgcatgccGAGGTGCAGCTGTTGCAGTCTGC 3’

BssHII-HuVH6aBACK: 5’ gCCgcgcgcatgccCAGGTACAGCTGCAGCAGTCAGG 3’

HuJH1–2FOR: 5’ TGAGGAGACGGTGACCAGGGTGCC 3’

HuJH3FOR: 5’ TGAAGAGACGGTGACCATTGTCCC 3’

HuJH4–5FOR: 5’ TGAGGAGACGGTGACCAGGGTTCC 3’

HuJH6FOR: 5’ TGAGGAGACGGTGACCGTGGTCCC 3’

Primers for hVH “nanobody” library

NheI-HuJH 1–2 FOR : 5’ CGGCCGCGCTAGCTGAGGAGACGGTGACCAGGGTGCC 3’

NheI-HuJH 3 FOR: 5’CGGCCGCGCTAGCTGAAGAGACGGTGACCATTGTCCC 3’

NheI- HuJH 4–5 FOR: 5’CGGCCGCGCTAGCTGAGGAGACGGTGACCAGGGTTCC 3’

NheI-HuJH 6 FOR: 5’CGGCCGCGCTAGCTGAGGAGACGGTGACCGTGGTCCC 3’

Primers for Vk

HuVκ1aBACK: 5’ GACATCCAGATGACCCAGTCTCC 3’

HuVκ2aBACK: 5’ GATGTTGTGATGACTCAGTCTCC 3’

HuVκ3aBACK: 5’ GAAATTGTGTTGACGCAGTCTCC 3’

HuVκ4aBACK: 5’ GACATCGTGATGACCCAGTCTCC 3’

HuVκ5aBACK: 5’ GAAACGACACTCACGCAGTCTCC 3’

HuVκ6aBACK: 5’ GAAATTGTGCTGACTCAGTCTCC 3’

NheI-HuJκ1FOR: 5’ CGGCCGCgctagcACGTTTGATTTCCACCTTGGTCCC 3’

NheI-HuJκ2FOR: 5’ CGGCCGCgctagcACGTTTGATCTCCAGCTTGGTCCC 3’

NheI-HuJκ3FOR: 5’ CGGCCGCgctagcACGTTTGATATCCACTTTGGTCCC 3’

NheI-HuJκ4FOR: 5’ CGGCCGCgctagcACGTTTGATCTCCACCTTGGTCCC 3’

NheI-HuJκ5FOR: 5’ CGGCCGCgctagcACGTTTAATCTCCAGTCGTGTCCC 3’

Primers for Vλ

HuVλ1BACK: 5’ CAGTCTGTGTTGACGCAGCCGCC 3’

HuVλ2BACK: 5’ CAGTCTGCCCTGACTCAGCCTGC 3’

HuVλ3BACK: 5’ TCCTATGTGCTGACTCAGCCACC 3’

HuVλ3bBACK: 5’ TCTTCTGAGCTGACTCAGGACCC 3’

HuVλ4bBACK: 5’ CACGTTATACTGACTCAACCGCC 3’

HuVλ5BACK: 5’ CAGGCTGTGCTCACTCAGCCGTC 3’

HuVλ6BACK: 5’ AATTTTATGCTGACTCAGCCCCA 3’

NheI-HuJλ1FOR: 5’ CGGCCGCgctagcACCTAGGACGGTGACCTTGGTCCC 3’

NheI-HuJλ2-3FOR: 5’ CGGCCGCgctagcACCTAGGACGGTCAGCTTGGTCCC 3’

NheI-HuJλ4-5FOR: 5’ CGGCCGCgctagcACCTAAAACGGTGAGCTGGGTCCC 3’

Primers for Linkers

PlusLinker(G4S)3 : 5’ GGTGGAGGCGGTTCAGGCGGAG 3’

MinusLinker(G4S)3: 5’ CGATCCGCCACCGCCAGAGCCAC 3’

RHuJH1–2: 5’ GCACCCTGGTCACCGTCTCCTCAGGTGG 3’

RHuJH3: 5’ GGACAATGGTCACCGTCTCTTCAGGTGG 3’

RHuJH4-5: 5’ GAACCCTGGTCACCGTCTCCTCAGGTGG 3’

RHuJH6mod: 5’ GGACCACGGTCACCGTCTCCTCAGGTGG 3’

RHuVκ1aBACKFv: 5’ GGAGACTGGGTCATCTGGATGTCCGATCCGCC 3’

RHuVκ2aBACKFv: 5’ GGAGACTGAGTCATCACAACATCCGATCCGCC 3’

RHuVκ3aBACKFv: 5’ GGAGACTGCGTCAACACAATTTCCGATCCGCC 3’

RHuVκ4aBACKFv: 5’ GGAGACTGGGTCATCACGATGTCCGATCCGCC 3’

RHuVκ5aBACKFv: 5’ GGAGACTGCGTGAGTGTCGTTTCCGATCCGCC 3’

RHuVκ6aBACKFv: 5’ GGAGACTGAGTCAGCACAATTTCCGATCCGCC 3’

RHuVλBACK1Fv: 5’ GGCGGCTGCGTCAACACAGACTGCGATCCGCCACCGCCAGAG 3’

RHuVλBACK2Fv: 5’ GCAGGCTGAGTCAGAGCAGACTGCGATCCGCCACCGCCAGAG 3’

RHuVλBACK3aFv: 5’ GGTGGCTGAGTCAGCACATAGGACGATCCGCCACCGCCAGAG 3’

RHuVλBACK3bFv: 5’ GGGTCCTGAGTCAGCTCAGAAGACGATCCGCCACCGCCAGAG 3’

RHuVλBACK4Fv: 5’ GGCGGTTGAGTCAGTATAACGTGCGATCCGCCACCGCCAGAG 3’

RHuVλBACK5Fv: 5’ GACGGCTGAGTCAGCACAGACTGCGATCCGCCACCGCCAGAG 3’

RHuVλBACK6Fv: 5’ TGGGGCTGAGTCAGCATAAAATTCGATCCGCCACCGCCAGAG 3’

**Datasets**

The fastq datasets and the associated errors are available at <http://laboratoriobiologia.sns.it/DEAL>

**Statistical analysis: Estimation of library complexity**

The theoretical complexity of the hScFv1, hScFv2 and hVH nanobody libraries has been estimated using the Negative Binomial (NB) distribution function, by a non-linear regression of the three scatter plots shown in Fig 4. The number of sequences (Nseq) is a function of cluster cardinality x, which is by definition an integer variable with values x={x_1_,…,x_N_} where x_i_>0 for measured data and x_N_ is the maximum cluster cardinality measured in the sequenced data. NB_p,s_(x) is defined as a discrete Poisson-Gamma mixture probability distribution of the x random variable, depending on two real parameters: the probability p, with p ∊ (0,1), and the size s, with s>0. To estimate the total library complexity C we assumed that Nseq follows a NB-like function of this form:  Nseq(x)~ C*NB_p,s_(x) where x is the cluster cardinality and p,s,C must be estimated from the experimental data.
The experimental value Nseq(x=0) does not exist and would account for the amount of sequences that were not  detected by the sequencing analysis, therefore this value can only be estimated from the regression function.
Since NB_p,s_(x) is defined for any x≥0, but library data are available only for a finite integer set of cardinality values x={x_1_,…,x_N_} where x_i_>0, we used for regression a truncated NB (tNB) distribution [4], defined on a subset of cardinality values, composed by the  whole integer interval I_m,n_={m,m+1,…,m+n-1,n} such that {m,…,n}⊂{x_1_,…,x_N_} and m>0.  tNB_p,s_(x) is a probability function defined zero outside I_m,n_, and 1/NormF* NB_p,s_(x) for x ∊ I_m,n_, where NormF is the Normalization factor to rescale the cumulative sum of the tNB function to 1. NormF is therefore the sum of NB_p,s_(x) across all values x ∊ I_m,n_. The data to be fit are transformed into an empirical truncated frequency distribution y(x) defined in the same interval x ∊ I_m,n_:  y(x)=1/NormS*Nseq(x), where NormS is the sum of Nseq(x) data across the interval and y(x)=0 outside the selected interval. The regression algorithm minimizes the Euclidean distance ||y(x) - tNB_p,s_(x)||^2^  for any x ∊ I_m,n_, thus estimating  p and s parameters of the NB function. C is estimated as the ratio between the sum of the data and the cumulative probability  in the interval:  C=Sum_x_(Nseq(x))/Sum_x_(NB_p,s_(x)) for x ∊ I_m,n_  [5]. The minimization was computed with data in Log_10_ scale for hscFv1 and hVH libraries, and with data in linear scale for the hscFv2. The interval I_m,n_  was [3,50] for hscFv, [4,55] for hscFv2 and [5,61] for hVH to allow algorithm convergence and avoid overestimation. The fit for the three libraries is shown in S3 Fig.

**Details of regression method for the nVH library**

We provide here, as a clarifying example, additional technical details for the regression of nVH library distribution. The data for the analyzed library are provided in the following 2-columns (x,y) table form:

| **x** | **y** |
| --- | --- |
| 1 | 3453641 |
| 2 | 516123 |
| 3 | 232665 |
| 4 | 136637 |
| 5 | 88197 |
| 6 | 61504 |
| 7 | 45145 |
| ... | … |
|  |  |
| 302 | 3 |
| ... | … |
| ... | … |
| 3388 | 1 |
| 3439 | 1 |
| 3979 | 1 |
| 5046 | 1 |
| 6778 | 1 |
| 19993 | 1 |

The table indicates that there are 3453641 sequences present in just one single copy, 516123 sequences in 2 copies, 232665 in 3 copies, and so forth up to one species in 19993 copies. P(x) = y(x)/ sum_x_ [y(x)] represents the empirical frequency distribution of the library.

To obtain the negative binomial fit we used for regression the truncated negative binomial distribution tNB_p,s_(x), defined in a limited interval x∈I_5,61_={5,6,…,60,61}. This is a probability function defined zero outside the interval, normalized by the sum of NB_p,s_ (x) across the whole I_5,61_  to rescale the integral of the distribution to 1. The boundaries of the interval were chosen to improve the fit to the NB distribution: i) discarding x={1,2,3,4} avoids overestimation, since these points are more error-prone; ii) x=61 is last point for which y(x)<y(x-1), guaranteeing that y(x) is a perfectly smooth decreasing function in the interval.

The parameter space to be explored for regression is the **R^2^** Cartesian subspace:

Par= {p} X {s} = [0 , 1] X [0 , 1] .

Indeed the algorithm samples the Par space n times, keeps the best 5% regression functions, shrinks the parameter space using the selected regressions, repeats the procedure until convergence.

Algorithm

*Preliminary_step*

Transform y data into truncated empirical probability distribution y_prob(x):

y_prob(x)=y(x)/ sum_x_ [y(x)] for x in the chosen interval I

y_prob(x)=0 for x outside I

Compute the squared deviation from the mean value in Log_10_ space, that will be used to evaluate fitting quality in each regression step.

SS_tot= sum_x_ [ ( log_10_ (y_prob(x)) -log_10_(y_mean(x)) )^2^ ]

*Step_1_*

Randomly sample in Log scale the Par space 2*10^4^ times, obtaining 2*10^4^ (p,s) couples of parameters.

Compute 2*10^4^ tNB_p,s_ (x) functions in the interval I.

Compute the classical fit goodness R^2^, using the Euclidean distance between the empirical data probability distribution and the tNB_p,s_ (x) fit functions

dd= sum_x_ [ ( log_10_ (tNB_p,s_(x)) -log_10_(y_prob(x)) )^2^ ]

R^2^=1 - dd/SS_tot

The more R^2^ approaches 1.0 , the better the regression

Select the best 5% fits, that is the 1000 tentative regression functions tNB_p,s_ (x) with the best fit goodness R^2^. From this selection, extract the minimum and maximum values for the parameters distribution. Thus the new parameter subspace to explore is now smaller than the original one:

Par = [min(p), max(p)] X [min(s) , max(s)]

*Step_n_*

Repeat Step n-1, sampling 2*10^4^ times the new smaller Par subspace

Select the best 1000 regressions and further shrink the parameter subspace

Evaluate the difference between the best R^2^  values in Step_n-1_ and Step_n_, using the median estimator:

Delta= median(R^2^_step_n-1_) - median(R^2^ _step_n_)

This difference decrease at every step, since R^2^ improves. The computation stops when R^2^ does not improve significantly any more. We chose to stop when Delta<10^-13^. This usually happens after about 18-20 steps.

*End_step*

When the computations stops, the estimated regression parameters are:

**s**_fit = median(best 1000 s parameters of last step) , **p**_fit = median(best 1000 p parameters of last step)

The estimated truncated negative binomial function is tNB_p_fit,s_fit_(x), which is the native NB_p_fit,s_fit_(x) but rescaled with the normalization factor before mentioned. The library complexity C is then estimated as the ratio between the sum of data and the cumulative probability of the negative binomial evaluated in the selected interval I.

1 / Sum_x_(NB_p_fit,s_fit_ (x)) = C / Sum_x_(Y(x)) 🡪 C=Sum_x_( Y(x)) /Sum_x_(NB_p_fit,s_fit_ (x)) , with x ∈ I

To evaluate the regression and convergence quality, we repeated this process about a hundred times and computed the standard deviation of the complexity estimates.

For the nVH library the diversity regression result was 6.41*10^6^ ± 18.

**Supporting Information References**

1. Marks JD, Bradbury A. PCR cloning of human immunoglobulin genes. Methods Mol Biol. 2004;248: 117–134. doi:10.1385/1-59259-666-5:117

2. Visintin M, Quondam M, Cattaneo A. The intracellular antibody capture technology: towards the high-throughput selection of functional intracellular antibodies for target validation. Methods. 2004;34: 200–214. doi:10.1016/j.ymeth.2004.04.008

3. Elsaesser R, Paysan J. Liquid gel amplification of complex plasmid libraries. Biotechniques. 2004;37: 200,202. doi:10.2144/3702A0200

4. Thygesen HH, Zwinderman AH. Modeling Sage data with a truncated gamma-Poisson model. BMC Bioinformatics. 2006;7: 157. doi:10.1186/1471-2105-7-157

5. Chao A, Lee S-M. Estimating the Number of Classes via Sample Coverage. J Am Stat Assoc. 1992;87: 210–217. doi:10.1080/01621459.1992.10475194
